# Aggregation and remineralization of *Trichodesmium* unveil potential for ocean carbon sequestration

**DOI:** 10.1093/ismeco/ycaf128

**Published:** 2025-07-29

**Authors:** Marion Fourquez, Fatima-Ezzahra Ababou, Mercedes Camps, France Van Wembeke, Olivier Grosso, Aude Barani, Sandra Nunige, Léa Guyomarch, Frédéric A C Le Moigne, Sophie Bonnet

**Affiliations:** Aix Marseille Univ., Université de Toulon, CNRS, IRD, Mediterranean Institute of Oceanography (MIO, UMR110), 13288 Marseille, France; Aix Marseille Univ., Université de Toulon, CNRS, IRD, Mediterranean Institute of Oceanography (MIO, UMR110), 13288 Marseille, France; Aix Marseille Univ., Université de Toulon, CNRS, IRD, Mediterranean Institute of Oceanography (MIO, UMR110), 13288 Marseille, France; Aix Marseille Univ., Université de Toulon, CNRS, IRD, Mediterranean Institute of Oceanography (MIO, UMR110), 13288 Marseille, France; Aix Marseille Univ., Université de Toulon, CNRS, IRD, Mediterranean Institute of Oceanography (MIO, UMR110), 13288 Marseille, France; Aix Marseille Univ., Université de Toulon, CNRS, IRD, Mediterranean Institute of Oceanography (MIO, UMR110), 13288 Marseille, France; Aix Marseille Univ., Université de Toulon, CNRS, IRD, Mediterranean Institute of Oceanography (MIO, UMR110), 13288 Marseille, France; Aix Marseille Univ., Université de Toulon, CNRS, IRD, Mediterranean Institute of Oceanography (MIO, UMR110), 13288 Marseille, France; Univ Brest, CNRS, IRD, IFREMER, Laboratoire des sciences de l’environnement marin, 29280 Plouzané, France; Aix Marseille Univ., Université de Toulon, CNRS, IRD, Mediterranean Institute of Oceanography (MIO, UMR110), 13288 Marseille, France

**Keywords:** diazotrophs, organic matter remineralization, Trichodesmium erythraeum, carbon sequestration, sinking velocity, aggregation, biological carbon pump

## Abstract

Recent studies have shown that diazotrophs can form aggregates sinking at velocities up to 400 m d^−1^, challenging the long-standing assumption that these organisms are confined to the surface ocean, and suggesting an understimated role in carbon (C) sequestration in warm oligotrophic waters. Yet, the extent to which their biomass escapes remineralization in the mesopelagic zone remains poorly constrained. Here, we experimentally simulated the aggregation and sinking of the filamentous diazotroph *Trichodesmium erythraeum* using roller tanks, following organic matter distribution over a 10-day period—equivalent to a 1000 m descent at a sinking velocity of ~100 m d^−1^. Our results show that 33% of organic C and 36% of N remained in the particulate fraction at the end of the experiment, indicating that microbial remineralization was incomplete and relatively proportional. Remineralization was most intense during the first 3 to 5 days of descent (0–500 m), after which potential C flux declined. We also estimated that a substantial portion of bacterial biomass was incorporated into the aggregates and may contribute to the C export, revealing a dual role for bacteria as both recyclers and exporters of organic matter. Given the widespread distribution and high productivity of *Trichodesmium erythraeum* in the expanding (sub)tropical ocean, our results highlight the need to include its fate in global biogeochemical models.

## Introduction

Diazotrophs are planktonic organisms that play a vital role in ocean ecosystems by supplying new nitrogen (N) through dinitrogen fixation (N₂ fixation). This biological process converts inert atmospheric N₂ into bioavailable forms (e.g. ammonia) and fuels most new primary production (PP) [1, 2] and export production in oligotrophic N-limited regions [3]. As oligotrophic gyres are projected to expand under future climate scenarios [4, 5], diazotrophs may be essential contributors to future net primary production (NPP) across these vast areas [6]. Reflecting their growing importance, climate models have begun to incorporate the role of diazotrophs in projecting future marine NPP. However, significant uncertainties remain, especially in tropical regions where diazotrophs thrive. Some models project decreases in global NPP [7]; others suggest increases due to divergent N_2_ fixation parameterizations [6, 8]. Regional uncertainties, particularly in the Indo-Pacific, pose challenges for assessing future ecosystem impacts and services in the ocean [9]. Factors contributing to these uncertainties include particle formation processes and remineralization rates [10]. These uncertainties largely stem from the limited understanding of the pathways through which diazotrophs contribute to carbon (C) downward export on a global scale.

Currently, two potential export pathways have been identified: direct and indirect export. The direct pathway involves the gravitational settling of diazotrophs to the deep ocean [11], while the indirect pathway transfers organic carbon (OC) derived from diazotrophs biomass through the food web [12, 13]—a process often referred to as the N₂-primed prokaryotic C pump. For both pathways, the amount of C derived from N₂ fixation that reaches the deep ocean depends on the sinking velocity of the particle carrying it and on the rate of remineralization it undergoes in the upper mesopelagic ocean [14]. These rates are influenced by various factors, such as the surface plankton community composition [15], mineral ballast [16–18], and temperature [19], among others. Remineralization by heterotrophic prokaryotes (hereafter “bacteria”) also largely accounts for the loss of exported particulate OC (POC) in the ocean [20, 21], although particle-attached microbes were found to account for 7%–29% of POC flux attenuation, suggesting that zooplankton play a more dominant role in this attenuation process [21].

This bacterial remineralization process converts OC into dissolved inorganic C (DIC) within the water column, helping to sustain the C concentration gradient between the surface and deep ocean [22]. Over time, lateral advection and vertical mixing transport DIC back to the surface, where it may ultimately be released into the atmosphere.

The time it takes for DIC to return to the surface is influenced by the depth at which remineralization occurs. Below 1000 meters (i.e. below the maximum depth of the permanent pycnocline of ca. 1000 m), physical processes can take from centuries to millennia to return DIC to the surface [22]. The direct export of nonballasted diazotrophs has long been assumed to be small and their fate limited to upper ocean layers where they were deemed to be fully remineralized [23]. This was especially the case for the filamentous cyanobacterium *Trichodesmium* is a senescent state. Although no longer actively regulating their buoyancy, they may still retain gas vacuoles, thus limiting its export to the deep ocean [24–26].

However, recent studies challenged this consensus by providing qualitative data revealing that *Trichodesmium* was detected below the euphotic zone, sometimes as deep as 4000 m, across the subtropical ocean [27–30]. Quantitative PCR on the *nifH* gene and N conversion factors [29] showed that *Trichodesmium* is massively exported and accounts for up to ~80% of the total particulate organic N (PON) export at 1000 m in the Western subtropical South Pacific ocean. Additionally, Benavides *et al.* [28] reported actively N_2_-fixing *Trichodesmium* colonies at depths of 1000 m, suggesting rapid vertical transport through sinking. This was further corroborated by Ababou *et al*. [31], who demonstrated that laboratory-generated *Trichodesmium* aggregates can sink at ~100 m d^−1^ or even faster when ballasted by dust or biological materials [32, 33]. Collectively, these studies suggest that *Trichodesmium* and other diazotrophs may be transported to the deep ocean more efficiently than previously assumed. Despite these advances, the fate of diazotroph aggregates within the microbial loop during their descent remains largely unknown.

In light of these recent findings, our goal in this study was to quantify the proportions of C and N derived from N₂ fixation that could potentially escape remineralization. We focused on *Trichodesmium*, a filamentous, colony-forming, and globally distributed marine diazotroph, typically exceeding 100 μm in size [34]. Aggregates formed by *Trichodesmium* have been shown to sink at sufficient velocities to escape remineralization in the upper ocean and reach mesopelagic or deeper layers [31]. Because measuring *in situ* remineralization rates of specific plankton species is challenging, we adopted an experimental approach.

*Trichodesmium* aggregates were formed in rotating cylindrical tanks following the method developed by Shanks and Edmonson [35]. This “roller tank” approach allows particles to sink continuously as they would in the water column, facilitating aggregation primarily by physical processes. We applied this method to simulate the sinking of *Trichodesmium* aggregates to a depth of 1000 m. Through this experimental setup, we aimed to quantify the proportion of C and N in *Trichodesmium* biomass that undergoes remineralization during the sinking process, providing insights into its potential contribution to C sequestration in the deep ocean.

## Materials and methods

### Experimental set-up

**Figure 1:**
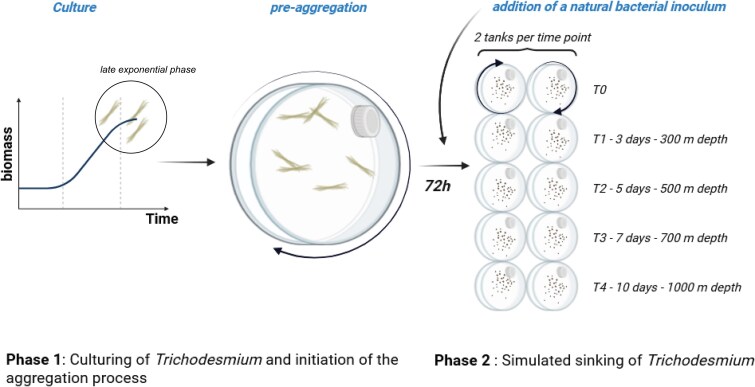
Experimental set-up.

The filamentous diazotroph *Trichodesmium erythaeum* (strain IMS101, hereafter referred to as *Trichodesmium*) was used to artificially form aggregates in roller tanks. Throughout the experiment, we monitored several biological and chemical parameters (described hereafter) to monitor the dynamics of remineralization. The experiment was divided into two phases: (i) Phase 1 corresponded to the formation of aggregates for a period of 72 h based on our previous study [31], and (ii) Phase 2 of the experiment lasted 10 days, simulating a sinking depth of ~1000 m, based on an estimated sinking velocity of ~100 m d^−1^ [31]. Visually, aggregation has started after 72 h in the tanks (referred to as time 0, T0). The second phase of the experiment lasted 10 days. The experimental setup is presented in Fig. 1.

#### Phase 1: Culturing of *Trichodesmium* and initiation of the aggregation process

*Trichodesmium* was grown at 27°C under a 12:12-h light–dark cycle with an irradiance of 100 μmol photons m^−2^ s^−1^. The culture medium consisted of autoclaved and 0.2 μm-filtered natural seawater (collected at 43°14′30″N, 5°17′30″E, Mediterranean Sea), enriched with nutrients in the same proportions as the YBCII medium [36]. The cultures were maintained in sterilized 10 liters (l) glass bottles until they reached the late exponential growth phase and then pooled into a 25 l autoclaved Nalgene carboy to ensure homogenization. At the end of exponential growth, cultures were transferred to 3.45 l rolling tanks filled to avoid air bubbles, enabling solid body rotation to keep particles suspended and mimic sinking in the water column [36]. Each tank contained 70% culture and 30% of sterilized medium identical to the original culture medium but without added nutrients. Immediately after filling the tanks, an initial sampling was performed to measure dissolved and particulate OC (DOC and POC, respectively), confirming consistent organic C levels across all tanks. The 10 tanks were then placed on a roller table in the dark at 23°C, simulating the temperature at the base of the photic zone in subtropical waters [3], where these diazotrophs naturally thrive. The tanks were rotated at three rotations per minute [31].

#### Phase 2: Simulated sinking of *Trichodesmium*

**Figure 2:**
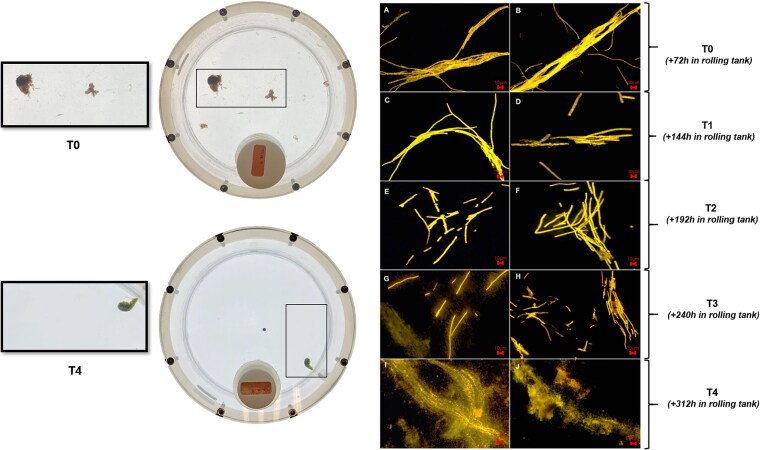
Epifluorescence microscopy images of *Trichodesmium* aggregates at different time points during Phase 2. (A, B) T0, (C, D) T1 (3 days after the start of Phase 2), (E, F) T2 (5 days after the start of Phase 2), (G, H) T3 (7 days after the start of Phase 2), and (I, J) T4 (10 days after the start of Phase 2).

Over the 72-h period, visible macroscopic aggregates formed (Fig. 2). Five sampling points were taken from T0 to T4, with two tanks (biological replicates) harvested at each time point (3, 5, 7, and 10 days). Subsequent sampling was made in triplicates (technical replicates) for the parameters described below.

### Inoculation of natural bacterial community

During the remineralization experiment, *Trichodesmium* aggregates were incubated with bacteria. Following the phase 1 (Fig. 1), the 10 tanks were opened under a laminar flow hood and exposed to ambient air for 1 h to allow for re-oxygenation of the water. A natural inoculum of bacteria was then added to the tanks as follows: 100 ml of seawater from each tank were carefully removed from the surface (avoiding aggregates) and replaced by 100 ml containing 3.4 × 10^6^ bacteria ml^−1^. The bacterial inoculum was prepared by collecting 4 l of natural seawater (43°14′30″N; 5°17′30″E, 7 m depth, Mediterranean Sea) filtered in sequence on 0.8 and 0.2 μm polycarbonate filters. This allowed for pre-concentrating free living bacteria (0.2–0.8 μm), and the resulting bacterial biomass was resuspended in 1.1 l of the same 0.8 μm-filtered seawater 2 h after collection. After bacterial inoculation, the tanks were closed and placed on the roller table at 20°C in the dark. This point corresponds to the time zero (T0) of Phase 2 of the experiment (Fig. 1); two tanks were immediately harvested for measuring the parameters cited below (see Sampling Procedures section).

### Sampling procedures

During Phase 2, following the beginning of aggregation, two distinct fractions formed: the aggregate fraction (AGG) and the water fraction (W) containing non-aggregated cells. Both fractions were collected at each time point, except at T0, where only the bulk was considered to avoid sampling bias, as many non-aggregated trichomes were still floating on the surface of the tanks. At each time point, two tanks were removed from the roller table and once the aggregates settled, the tanks were opened under a laminar flow hood for sampling. Visible aggregates were carefully collected using a 50 ml pipette (wide mouth to avoid damaging aggregates) and dispensed into a precombusted glass bottle with 0.2 μm-filtered, C- and N-free seawater to reach a final volume of 400 ml. This preparation of a diluted `slurry' provided sufficient volume to collect subsamples for the following analysis: POC, PON, epifluorescence microscopy, and heterotrophic prokaryotic production (BP, for bacterial production). Results expressed on a volumetric basis account for this dilution. Once the tanks were freed of visible aggregates, sampling in the W fraction was performed for the same parameters as for aggregates plus DOC, nitrate + nitrite (NO_x_) and phosphate (PO_4_^3−^) concentrations and free-living bacterial abundance.

### Observations and analyses

#### Qualitative observation of *Trichodesmium* aggregates by epifluorescence microscopy

For the W and AGG fractions, 20–50 ml were collected onto 25 mm diameter, 2 μm polycarbonate membranes and fixed with 2% paraformaldehyde diluted in filtered seawater for 15 min and stored at −20°C until observation. Photography were taken using an epifluorescence Zeiss Axio Imager II microscope equipped with a mercury lamp (HBO 100W) mounted with a dichroic filter of 545 ± 25 excitation (ex.) and 605 ± 70 emission (em.) for phycoerythrin (orange fluorescence).

#### Dissolved organic C and particulate organic C and N

DOC samples were collected from duplicate tanks in three technical replicates. These samples were filtered under a laminar flow hood through a 0.2 μm Sartorius cartridge connected to a peristaltic pump and transferred into precombusted (450°C, 4 h) 20 ml glass vials. To preserve the samples, 20 μl of 96% sulfuric acid (H_2_SO_4_) were added, and they were stored at 4°C in the dark until analyzed with a Shimadzu TOC-V analyzer [37]. POC and PON samples collected from the W and AGG fractions in the duplicate tanks and in three technical replicates were filtered onto precombusted (450°C, 4 h) GF/F filters and dried at 60°C before being analyzed with an elemental analyzer coupled to an isotope ratio-mass spectrometer (EA-IRMS, Integra 2) according to Bonnet *et al*. [38].

#### Inorganic nutrients

Samples for the determination of NO_x_ and PO_4_^3−^ concentrations were collected from duplicate tanks in three technical replicates from the W fraction. As for DOC samples, they were filtered and transferred to acid-washed 20 ml polyethylene flasks and stored at −20°C until analysis using a segmented flow analyzer. Limitis of quantification were 50 and 20 nM for NO_x_ and PO_4_^3^, respectively [39].

#### Bacterial production and abundance

BP was estimated from protein synthesis rates via ^3^H-leucine incorporation using the microcentrifugation technique [40], as detailed in [41]. Briefly, subsamples of 1.5 ml were incubated in the dark at 20°C for 0.15–2 h. Leucine was added at a total concentration of 23 nM in THE W fraction samples and 54 nM in the AGG fraction, using a mix of cold and radioactive leucine with a specific activity of 100 Ci mmol^−1^ (curie per millimole). The linearity of leucine incorporation with time and the isotopic dilution were verified periodically through time series and concentration kinetics. A leucine-to-C conversion factor of 1.5 kg C mol^−1^ was used [42].

Bacteria were enumerated for the W fraction only by flow cytometry. For each time point, 4 ml of water samples were collected, fixed with paraformaldehyde (2% final concentration) and stored at −80°C. For analysis, samples were slowly thawed at room temperature, filtered through 20 μm pore-size filters, and stained with SYBR Green for 15 min in the dark [43]. TruCount beads (BD Biosciences®) and 2 μm beads (Fluoresbrite YG, Polysciences) were added to the samples as internal standards for cell concentration determination and size estimation. Samples were acquired using a Cytoflex Legacy analyzer (Beckman Coulter) of the PRECYM flow cytometry platform (https://precym.mio.osupytheas.fr/), equipped with three solid-state lasers (488, 405, and 638 nm) and a peristaltic pump system that allows to access to the absolute volume analysed. Data were acquired in log scale and stored in list mode then data analysis for bacterial enumeration was performed *a posteriori*, both using the CytExpert software (Beckman Coulter).

Cell-specific BP was calculated for the W fraction by dividing total BP by the cell count, yielding the production rate per individual cell [44, 45].

#### Statistical analyses

At each time point, the experiment included two tanks serving as biological replicates. Due to the limited number of biological replicates (*n* = 2 per time point), statistical comparisons were not performed. Instead, trends are interpreted descriptively with the mean of the two biological replicates at each time point. The standard deviation (SD) is also reported to reflect the variability between biological replicates (*n* = 2), except at T0, where 10 biological replicates were available (*n* = 10).

## Results and discussions

### Carbon and nitrogen budgets

**Figure 3:**
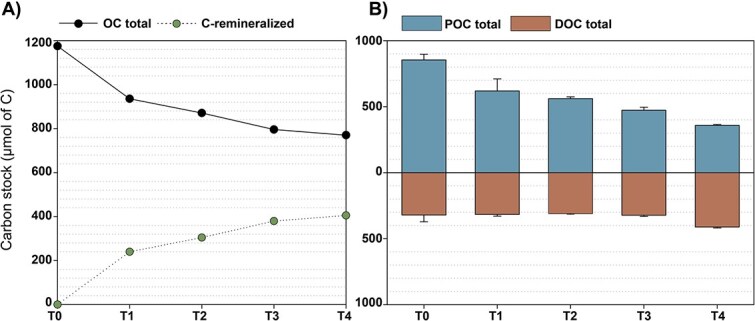
Evolution over time of (A) total organic carbon (OC) stock and estimated C-remineralized, calculated from the difference between the OC stock at T0 and the stock at each subsequent time point (T1–T4) and (B) stocks of dissolved OC (DOC) and particulate OC (POC). Bars and error bars represent the mean and standard deviation of two tanks (biological replicates), respectively.

At the start of the experiment, the average DOC and POC stocks in the tanks were 422 ± 55.4 μmol C and 843 ± 44.6 μmol C, respectively (*n* = 10), indicating consistent and reproducible OC levels across all replicates (Fig. 3). When aggregates began to form (T0—Phase 2), DOC stocks had decreased to 321 ± 52.0 μmol C, likely due to bacterial consumption of DOC associated with *Trichodesmium* cultures (microbiome) during Phase 1. Following the addition of the natural bacterial consortium at T0, DOC levels remained relatively stable during the first 7 days (T3) and averaged 317 ± 21.3 μmol C but then increased by the final time point (T4) to reach 412.2 ± 7.3 μmol C (Fig. 3).

In contrast, by the end of the experiment, POC stocks had decreased by 58 ± 0.82% and went from 854 ± 42.8 μmolC (*n* = 10) to 358 ± 7.01 μmolC (*n* = 2) between T0 and T4 (Fig. 3). This is consistent with the visual inspections of *Trichodesmium* indicating the progressive degradation of cell integrity over time, with colonies being gradually reduced to individual filaments and often smaller trichomes (e.g. T2–T3, Fig. 2). By the end of the experiment, the trichomes appeared highly burst (T4, Fig. 2). Closed systems, such as tanks, offer the advantage of allowing comprehensive C budget assessments, as there is no possible external input or loss of C. Based on the initial OC stock (POC + DOC at T0), 34.5% of the total OC stock was lost over the 10-day duration of Phase 2. This loss of C, observed as the difference between the initial and final OC stock, was attributed to C remineralization driven by bacterial activity (C-remineralized, Fig. 3).

**Figure 4:**
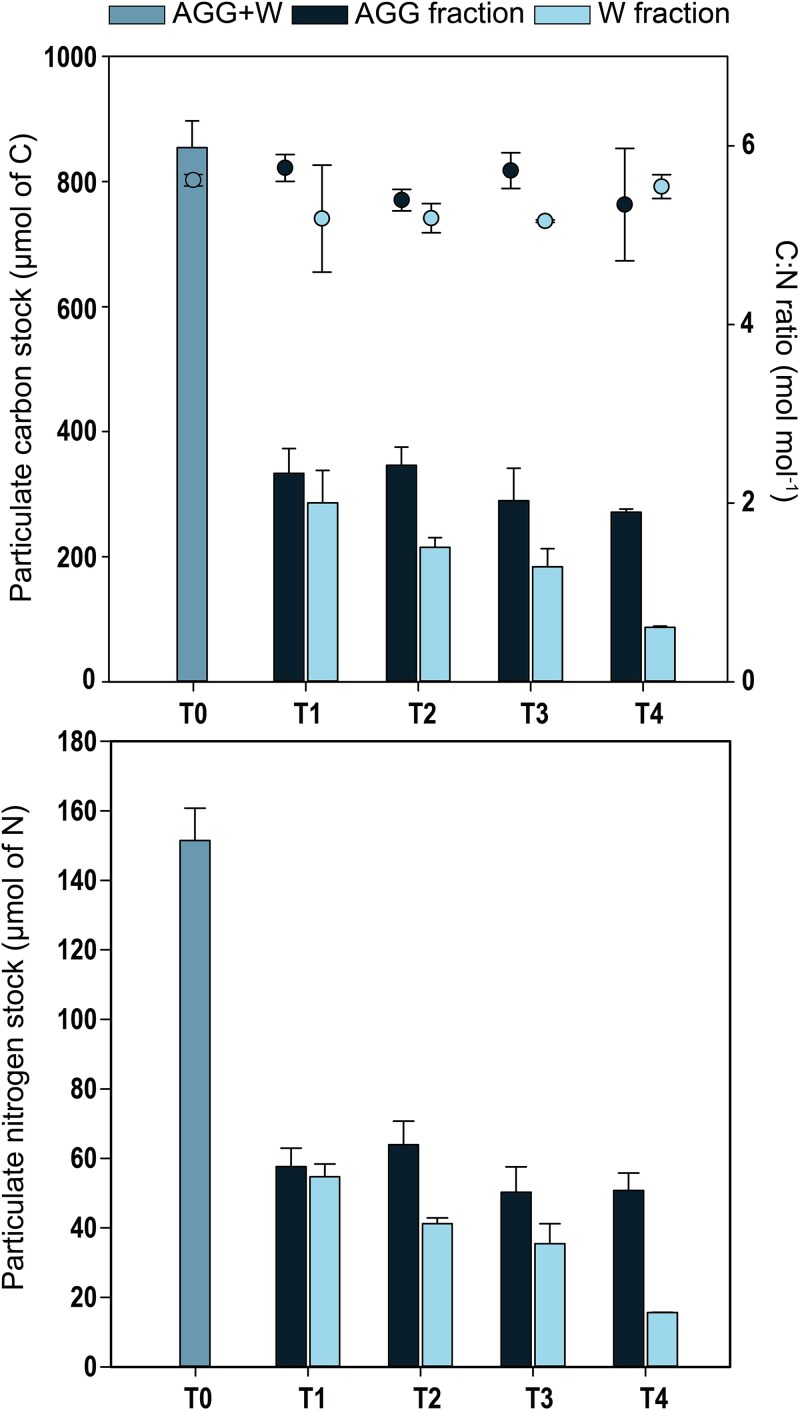
Stocks of particulate organic carbon (POC, top) and nitrogen (PON, bottom) during Phase 2 of the experiment. The two fractions are represented: aggregates (AGG) and water (W) together with the POC/PON ratio (C:N ratio, dots, top chart). Error bars represent the standard deviation of two tanks by time point except for T0 (*n* = 10).

The partitioning of POC and PON between W and AGG fractions provides insights into the process of aggregation. After 3 days (T1), which corresponds here to a sinking depth of 300 m, we observed that 26 ± 0.5% of POC had been transferred from the W fraction to the AGG fraction. This is slightly higher—but within the same range—as previously reported (18 ± 6% at ~400 m, [31]). Our study takes it a step further by showing a 90% in POC levels in the W fraction from T0 to T4 (simulated depth of 1000 m) which highlights a greater aggregation capacity than previously observed. As for POC, the average PON stocks at the start of the experiment (W + AGG fraction) were consistent across the 10 tanks (149.0 ± 9.8 μmol of N, *n* = 10, T0). From T1 to T4, PON stocks in the W fraction decreased from 54.7 ± 3.65 to 15.6 ± 0.03 μmol N (Fig. 4), while in the AGG fraction, PON only decreased from 57.6 ± 5.34 to 50.7 ± 5.01 μmol N during these 7 days. Overall, PON stocks declined by >70% in the W fraction, compared to a 12% decrease in the AGG fraction (Fig. 4).

These trends in both POC and PON stocks were closely aligned, leading to fairly stable C:N ratios in both fractions. The initial C:N ratio (mol mol^−1^) was 5.64 ± 0.06 (*n* = 10), consistent with Redfield’s stoichiometric ratio. Variations in C:N ratios between T1 and T4 were generally low, but a slight decrease was observed in the W fraction between T0 and T3 with the C:N ratio dropping to 5.19 ± 0.01. This decrease was driven by a larger decline in POC compared to PON stocks. However, by T4, this variation had disappeared and the C:N ratio returned to 5.57 ± 0.13 molC mol N^−1^ (Fig. 4). If we simply consider the difference between the initial and final POC and PON (W + AGG fractions), then 58% and 56% of the POC and PON disappeared over the 10 days of the experiment, respectively. However, this apparent loss does not only reflect the remineralization of *Trichodesmium* biomass alone, as part of the organic C and N was likely incorporated into newly produced bacterial biomass over time. This newly generated bacterial biomass (heterotrophic C-produced) should be accounted for when interpreting the net loss of POC and PON. To investigate the proportion of *Trichodesmium* that was effectively remineralized during its simulated sinking to 1000 m, we subsequently analyzed the partitioning of POC in relation to bacterial abundances and activities.

### Bacterial cell abundance and production

**Figure 5:**
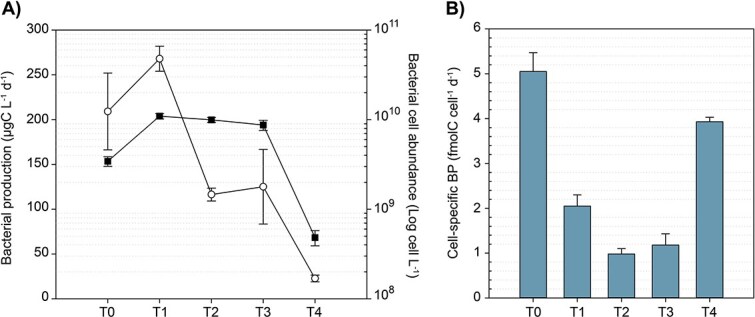
Evolution of (A) volumetric bacterial production (BP, dots) and (B) cell-specific BP over time during the simulated sinking of *Trichodesmium* in the W fraction. Bacterial cell abundances (squares) are shown in (A). Values represent the average of two tanks per time point for bulk BP (W + AGG fractions), with associated error bars representing standard deviation (SD).

During Phase 1, *Trichodesmium* colonies were only exposed to their microbiome. We compared BP and abundance before and immediately after the introduction of the natural consortium (Phase 2). If we consider the immediate increase of cell number, the natural bacterial consortium represented only 20% of total bacterial abundance at the beginning of Phase 2 (data not shown). In addition, at T0, only a 2% increase in total BP was measured immediately following the addition. Therefore, >98% of BP was due to the microbiome associated with *Trichodesmium* (data not shown) likely due to the time required for the natural bacterial consortium to acclimate. To clarify our findings, we first present the BP measured in the W fraction altogether with bacterial cell abundances (Fig. 5). Then we distinguish between BP measured in the W fraction and that in the AGG fraction.

In the W fraction, bacterial cell concentration increased 3.2-fold between T0 and T1, then remained relatively stable from T1 to T3, before sharply declining by T4 (Fig. 5). By Day 10 (T4), bacterial cell concentration was 7-fold lower than at T0 (3.4 × 10^6^ cells ml^−1^) and 23-fold lower compared to the peak abundance recorded on T1 (1.1 × 10^7^ cells ml^−1^). The BP followed this trend with an increase of activity recorded between T0 and T1 and dropped by 2.3 times between T1 and T2. Concurrently with the decrease in cell abundance, the lowest BP rates were measured at T4. As a result, cell-specific BP between T0 and T1 dropped due to an increase in cell abundance, indicating active cell division and growth during the Phase 2. By the end of the experiment, cell-specific BP rose from 1.18 ± 0.25 fmolC cell^−1^ d^−1^ at T3 to 3.93 ± 0.10 fmolC cell^−1^ d^−1^ at T4 (Fig. 5B). However, this increase reflects more the decline in free-living bacterial abundance than a rise in activity. Yet, it also suggests that the remaining cells in the W fraction were still metabolically active but that the growth conditions (i.e. lability of organic matter) or bacterial diversity may have shifted.

**Figure 6:**
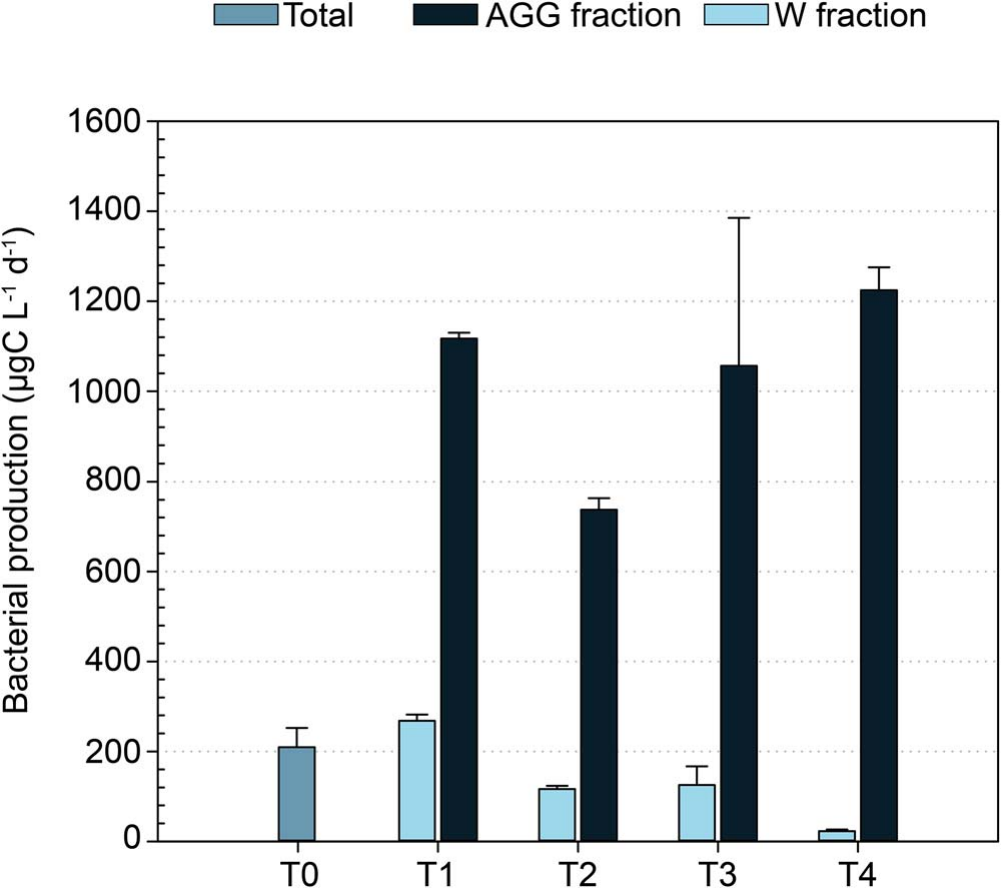
Volumetric bacterial production (BP) in the water fraction (W) and in aggregates fraction (AGG). Initial bacterial production (Total) at T0. Values represent the average of two tanks (biological replicates) per time point for BP with associated error bars representing standard deviation (SD) except for T0 (*n* = 10).

T0 marks the beginning of Phase 2, when aggregates started forming and the natural bacterial consortium was added. Comparing total BP (W fraction + AGG fraction) at T0 (209 ± 42.8 μgC L^−1^ d^−1^, *n* = 10) with BP measured in the AGG fraction, we observed a strong increase in bacterial activity starting at T1 (1117 ± 12.8 μgC L^−1^ d^−1^, *n* = 2), which remained elevated throughout the experiment (Fig. 6). This rise in BP likely indicates rapid colonization of the *Trichodesmium* aggregates by the natural consortium, which initially made up just 20% of the cell abundance at the start of Phase 2. This trend, along with the increase in dissolved inorganic phosphorus concentration (Supplementary Materials, serving as a proxy for remineralization), confirms that the remineralization of organic matter took place in the tanks. By assuming that C-remineralized corresponds to the release of C into the DIC pool due to bacterial C respiration (BR), and considering that newly formed organic C is produced through BP, we can estimate bacterial growth efficiency (BGE) as BGE = BP/(BP + BR). The results showed BGE values ranging from 0.24 to 0.43 (Table 1), indicating varying efficiencies in C utilization over the course of the experiment. Notably, the higher BGE of 0.43 observed during the first 5 days (T1 and T2) of Phase 2 suggests that nutrient availability effectively sustains high microbial efficiency.

**Table 1:** Total (W + AGG fractions) heterotrophic carbon produced and remineralized, and bacterial growth efficiency (BGE) for each time point.

	Heterotrophic C-produced (μmolC)	C-remineralized (μmolC)	BGE
T1	178	240	0.43
T2	228	304	0.43
T3	118	379	0.24
T4	214	405	0.35

**Figure 7:**
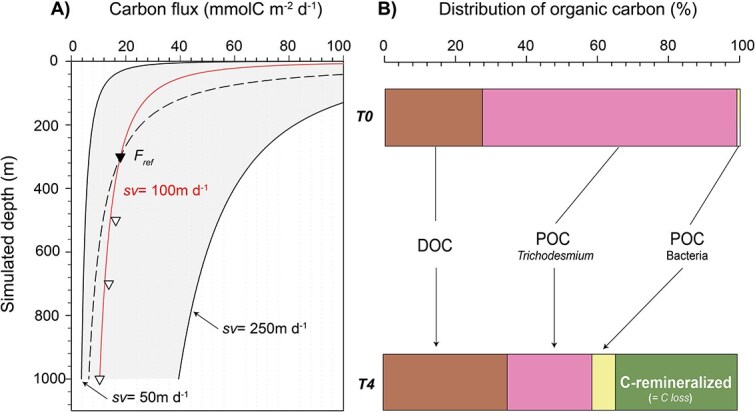
Evolution of the C stock over the time course of the experiment. (A) Estimated flux of C in mmolC m^−2^ d^−1^. Triangles represent flux at given depth, ${F}_z$, derived from this data set with ${F}_z={POC}_z\times sv$, using a fixed sinking velocity (sv) of 100 m d^−1^. The inverted triangle indicates the reference depth (300 m) with a corresponding flux ${F}_{ref}=$ 18 ± 2.3 mmolC m^−2^ d^−1^ (*n* = 6; three technical replicates for each tank and two tanks per simulated depth); Martin curves were derived with ${F}_z={F}_{ref}\times{\left(\frac{Z}{Z_{ref}}\right)}^{-b}$. The “*b*” value was estimated by fitting the log-transformed data to Martin’s curve using the following formula: $b=\frac{\mathit{\ln}\left({F}_{ref}/{F}_z\right)}{\ln \left(Z/{z}_{ref}\right)}$. An attenuation coefficient “*b*” = 0.45 was found to describe the average attenuation of POC flux using the present dataset (red line). For comparison, the dashed line used the original Martin’s factor (*b* = 0.86). The gray area represents an uncertainty range, using sv = 50 and 250 m d^−1^ to better reflect the inherent variability of carbon flux. (B) Proportion of organic C including estimates of bacterial (POC bacteria) and *Trichodesmium* (POC *Trichodesmium*) biomasses. Proportion of each fraction, dissolved organic C, POC-*Trichodesmium* and POC-bacteria were calculated at T0 and T4 as follows: Stock of C /total C stock × 100. C-remineralized was proportionally deduced from the OC loss between T0 and T4.

It is worth noticing that a more intense remineralization occurred during the first 5 days of simulated sinking, corresponding to depths of 0–500 m. This was supported by observed trichome degradation, colonies disaggregation (Fig. 2), and increases in bacterial abundance and phosphate concentrations between Days T0 and T3 (Supplementary Materials S1). These findings are consistent with Martin’s empirical relationship, which states that 50% of POC exported from the photic zone is attenuated within the first 300 m of the water column [46]. The respiration of OC during vertical transport is often modeled by the Martin curve, a power law fitted to POC flux measurements [46]. In this model, the remineralization length scale increases with depth, meaning either the remineralization rate slows, the sinking velocity increases, or both [47]. This power law implies that OC consists of compounds with varying lability and sinking velocities. Figure 7 illustrates the evolution of POC flux with depth, derived from our experimental dataset using a fixed sinking velocity of 100 m d^−1^. To better reflect the variability inherent to this parameter in natural settings, an uncertainty range is shown using bounding values of 50 and 250 m d^−1^ (Fig. 7A). This higher upper bound was chosen based on previous studies reporting sinking velocities exceeding 200 m d^−1^ for filamentous diazotrophs [31, 32] as well as on our recent findings from natural aggregates of *Trichodesmium* collected in the Pacific using the SNOWMAN (Simulator of Non-finite Open-Wheeled Marine Aggregation and siNking; Snowman Marine Instruments®, [48]). This graphical representation highlights both the sensitivity of C flux estimates to sinking velocity and the notable resemblance to Martin’s law. Figure 7 further emphasizes that microbial colonization likely plays a key role in shaping vertical flux attenuation by contributing additional C biomass to sinking particles (Fig. 7B). In particular, the colonization of *Trichodesmium* by bacteria raises important questions about how its microbiome might influence remineralization during sinking. More broadly, this points to a potentially overlooked influence of colonizing microorganisms on the sinking and degradation of particles. Could BP on aggregates play a more significant role in C sequestration than previously recognized?

### An overlooked role of bacterial production on aggregates in carbon sequestration?

BP measured using the ^3^H-leucine method reflects the C incorporated into biomass by active bacteria. In contrast, measurements of cell abundance provide an estimate of bacterial biomass (POC bact), which includes both active and inactive cells. This approach is more consistent when comparing POC bact with the POC total (W + AGG fractions), as the latter includes all particulate organic matter, regardless of metabolic activity or the state of the cells (living, dormant, or dead). Here, cell abundance was converted to C biomass using constant cell-to-C conversion factors based on the literature using 12.4 fg C cell^−1^ as reported by [49] Fukuda *et al*. (1998). However, bacterial cell abundance was directly measured by flow cytometry for the W fraction but not for the AGG fraction—as direct measurements were technically challenging. To estimate the bacterial cell abundance associated with the aggregates, we used a single linear regression based on the relationship between BP and cell abundance measured in the W fraction. This regression allowed us to recalculate the bacterial cell abundance attached to the aggregates from BP measured in the AGG fraction. From the following relationship, we thus estimated POC associated to bacteria in the W and the AGG fraction:


$$ {POC}_{Total}={POC}_{Tricho}+{POC}_{bact\ \left(W+ AGG\right)} $$

\begin{eqnarray*} \mathrm{With}\ {POC}_{bact\ \left(W+ AGG\right)} &=& \left( bacterial\ cell\ abundance\ per\ liter\right)\\ && \times 12.4\ fgC\ {cell}^{-1} \end{eqnarray*}

From this, we calculated the amount of POC associated with *Trichodesmium* by subtracting the portion attributed to bacterial biomass produced during the experiment (Fig. 7). By incorporating the POC derived from bacteria into our measurements, we determined that 67% of the POC originating from *Trichodesmium* was consumed, meaning that 33% escaped remineralization during its simulated descent to a depth of 1000 m. The difference between 67% and our initial estimate of 58% arises because that value was calculated as the difference between the initial and final total POC, without accounting for the C produced by bacteria over time. Although this bacterial-derived POC (heterotrophic C-produced) did not fully compensate for the overall loss of total POC, we estimated that bacterial POC accounted for nearly 22% by T4, compared to just 1.4% of the POC at T0 (Fig. 7). It is important to note, however, that our estimation may be subject to biases due to uncertainties in the quantification of bacterial cell abundance and the derived C-biomass. As such, we recommend that future studies aim to directly quantify the amount of POC produced by bacteria during similar experiments, in order to refine the amount of heterotrophic C-produced and its impact on POC dynamics during particle flux experiments.

Although bacteria are best known to remineralize OC and release CO₂, they may also contribute to the vertical export of C by being incorporated into sinking aggregates, thereby enhancing C sequestration. This dual role—where bacteria both attenuate and contribute to C export—raises the question of whether bacterial remineralization continues in the deep ocean or ceases at certain depths due to environmental conditions. Recent studies [50] found that bacterial respiration was lower on fast-sinking particles (>100 m d^−1^) than slow-sinking ones, suggesting that sinking velocity is an important factor of control for the remineralization process, but the specific contribution of bacterial biomass within aggregates to C export remains largely underexplored. One study [51], however, reported that bacterial biomass constituted 27% and 39% of the suspended particulate organic matter in surface waters of the oligotrophic Western Pacific, highlighting the potentially significant role of bacteria in C export processes. Therefore, incorporating growing bacterial biomass into models may result in higher estimates of global organic C sequestration compared to approaches using uniform remineralization. In addition, a simple comparison between C:N ratios in the particulate organic matter in the tanks (5.2–5.6) and the C:N ratio for marine bacteria (ranging from 5.0 to 8.3, ref [50] suggests that bacteria were not limited by either element. This is further reflected by the similar proportions of C and N derived from *Trichodesmium* biomass that were remineralized (Fig. 4).

### Technical limitations

It is important to acknowledge the limitations of using laboratory cultures to estimate remineralization rates for ecological interpretations, as these conditions may not fully reflect *in situ* dynamics. Controlled experiments, while valuable for isolating specific mechanisms, do not capture key environmental factors such as pressure variations and the dynamic shifts in bacterial communities that occur in natural settings. Nonetheless, such experiments provide critical insights that help to better understand the underlying processes governing remineralization.

Remineralization is driven by a combination of biological, chemical, and physical factors, which together determine the rates at which C, N, and other nutrients are recycled. The metabolic activity of microbial communities plays a key role in these rates, while the chemical composition of organic matter is equally important [52, 53]. This raises an interesting question about the lability of *Trichodesmium*, as the stoichiometry of organic material influences nutrient availability for bacteria. For instance, high C:N ratios can slow remineralization due to N limitation [54–56]. Here, the C:N ratio remained stable over the time course of the experiment, suggesting that the initial composition of *Trichodesmium* aggregates supports balanced C and N remineralization. In natural environments, shifts in the C:N ratio may also arise from mineral ballasting of sinking particles—such as those influenced by dust deposition—which can enhance sinking velocities and ultimately C sequestration efficiency [33, 57, 58]. The roller tank system was specifically designed to promote aggregation in a low-turbulence, grazer-free environment, thereby minimizing fragmentation caused by sloppy feeding, swimming activity, and microbial gardening [59, 60]. While fragmentation is a known factor contributing to flux attenuation in natural environments [36, 61], its influence was not observed and could not be quantified in our study. Therefore, we hypothesize these fast-sinking particles could reach the deep ocean with minimal degradation due to the short time in which they are available for fragmentation [17]. Similarly, although sinking velocity can vary widely *in situ* depending on particle size, porosity, and composition [16, 62, 63], deriving realistic estimates for non-ballasted particles such as *Trichodesmium* based solely on image-derived size remains challenging. Studies have shown that size-based predictions often underestimate or overestimate true sinking speeds, especially for filamentous or porous aggregates [62, 63]. Our chosen value of 100 m d^−1^ [31] thus reflects a conservative estimate, supported by recent observations of *Trichodesmium* aggregates showing velocities of 250 m d^−1^ in the natural environment and up to 500 m d^−1^ when ballasted with biological materials (unpublished data). This estimate is also consistent with the literature [32] measured sinking velocities of ~200 m d^−1^ and [33] measured rates ranging from 64 m d^−1^ (no dust) to 351 m d^−1^ (with dust). Finally, our roller tank experiments are conducted in closed systems, which may favor the development of an oxygen-depleted environment more readily than in the open ocean. In natural conditions, the degree of oxygenation surrounding aggregates could be higher due to water movement and exchange, potentially influencing the extent and rate of remineralization.

In our experimental tanks, PON concentrations were ~60 times higher than those observed during high-density *Trichodesmium* blooms measured during the TONGA campaign. Similarly, BP in our tanks was around 500 times greater than that measured in surface waters at the same site [64]. This substantial amount of organic matter availability likely stimulated bacterial activity in our experiments, as did the 20°C in the controlled room. However, given the greater bacterial activity also observed, it is likely that, under natural conditions, this organic matter would be remineralized even less efficiently than in our *in vitro* experiment. Yet, we are confident because our BGE estimates align with values reported in the Pacific Ocean, falling within the higher range of typically observed values (0.3–0.4), especially under conditions of high nutrient availability or in areas with intense upwelling [65, 66]. This experiment therefore represented an “ideal scenario” for bacteria to efficiently remineralize *Trichodesmium* aggregates, suggesting that our results likely underestimate the true export of C and N *in situ*. Given the estimated sinking velocity of *Trichodesmium* at ~100 m d^−1^, a significant portion of *Trichodesmium*-derived POC and PON could indeed reach depths >1000 m.

## Conclusion

This study was motivated by the intriguing discovery of well-preserved diazotrophs at depths below 1000 m, challenging the traditional belief that diazotrophs do not significantly contribute to C export through direct pathways. *Trichodesmium* blooms frequently occur in the (sub)tropical oceans and are accompanied by high C fixation and biomass production (26 000 mg C m^−2^ [67–69]), creating real oasis in the otherwise oligotrophic ocean. We confirmed previous findings that *Trichodesmium* colonies are capable of forming aggregates [31] and that only a fraction of it is effectively remineralized. Using N_2_ fixation rates of 52–73 Tg N year^−1^ [70] and assuming that 50% of the fixed N is retained as biomass while the remainder is released into surrounding waters as dissolved organic N or ammonium (as commonly observed in diazotrophs, e.g. [71]), *Trichodesmium*’s N contribution to biomass formation can be estimated between 26 and 36.5 Tg N year^−1^. Applying the Redfield ratio (C:N = 6.6:1), this translates into a C biomass production of ~171.6–240.9 Tg C/year. Therefore, if ~30% of this biomass is effectively exported to depth, *Trichodesmium* could contribute between 51.5 and 72.3 Tg C year^−1^ to deep ocean C sequestration. When compared to the global biological C pump flux of 0.2 Pg C year^−1^ (200 Tg C year^−1^) [6], *Trichodesmium* alone could account for ~25.8%–36.2% of the C exported to 1000 m depth annually—a substantial fraction for a single diazotrophic genus. However, when considering global estimates of *Trichodesmium* biomass ranging from 2 to 89 Tg C [69], *Trichodesmium*’s contribution to C export could range from 1% to 43% of the global BCP flux. This range underscores both the potential significance of *Trichodesmium* in oceanic C cycling and the need for further research to refine biomass estimates, export efficiencies, and N retention in different oceanic regions.

## Supplementary Material

_Supplementary_Materials_ycaf128

## Data Availability

All data generated or analysed during this study are included in this published article. Correspondence and requests for materials should be addressed to M. Fourquez.
