# Supplementary material for: Aggregation and remineralization of Trichodesmium unveil potential for ocean carbon sequestration
Source: ISME Commun. 2025 Jul 29;5(1):ycaf128. doi: 10.1093/ismeco/ycaf128 (PMC12448404; doi:10.1093/ismeco/ycaf128)
Supplement: _Supplementary_Materials_ycaf128 [file _supplementary_materials_ycaf128.docx]

# Supplementary Materials

*Supplementary Figure 1*

**
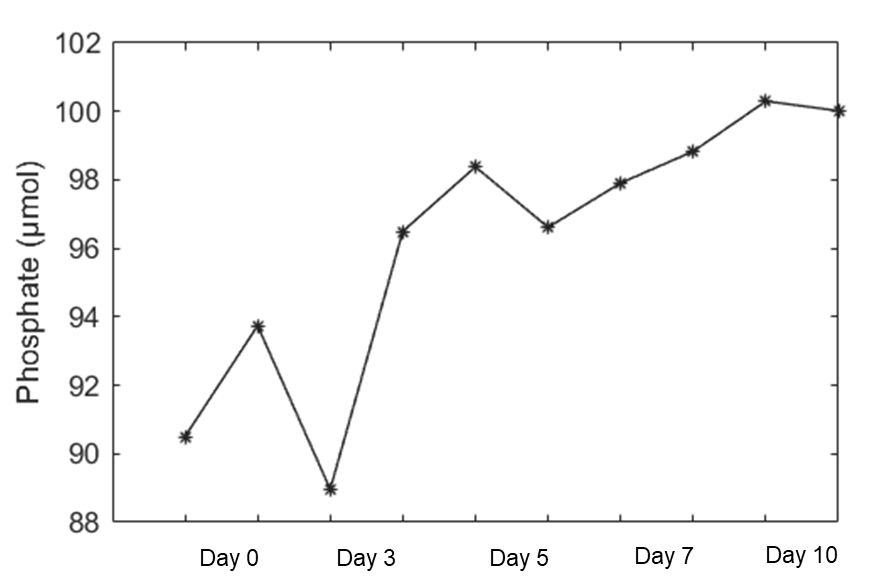
**

Stocks of phosphate in the tanks during the experiment. At each sampling point, two tanks were averaged: Day 0 (Tank 1 and Tank 2), Day 3 (Tank 3 and Tank 4), Day 5 (Tank 5 and Tank 6), Day 7 (Tank 7 and Tank 8) and Day 10 (Tank 9 and Tank 10).
